# Evaluation of the clinical characteristics and risk factors for in-hospital mortality amongst older persons admitted with bacteraemia in a geriatric ward – a single tertiary centre review

**DOI:** 10.1186/s12877-026-07141-2

**Published:** 2026-02-27

**Authors:** Nai Shan Yeat, Soo Tein Ngoi, Adrian Yen Xian Lee, Ruhana Che Yusof, Shailaja Sockaligam, Rosmahani Mohd Ali, Haiza Maisarah, Maw Pin Tan, Izzati Sae’ don, Kejal Hasmukharay

**Affiliations:** 1 Faculty of Medicine, Universiti Malaya, Kuala Lumpur, 50603 Malaysia; 2 Department of Anaesthesiology, Faculty of Medicine, Universiti Malaya, Kuala Lumpur, 50603 Malaysia; 3 Hospital Queen Elizabeth, Kota Kinabalu, Sabah 88200 Malaysia; 4 Faculty of Medicine, University of Cyberjaya, Selangor, 63000 Malaysia; 5 Hospital Kuala Lumpur, Kuala Lumpur, 50586 Malaysia; 6 Geriatric Unit, Hospital Kuala Lumpur, Kuala Lumpur, 50586 Malaysia; 7 Geriatric Unit, Department of Medicine, Faculty of Medicine, Universiti Malaya, Kuala Lumpur, 50603 Malaysia

**Keywords:** Older persons, Bacteraemia, Systemic inflammatory response syndrome (SIRS), In-hospital mortality

## Abstract

**Background:**

Bloodstream infections in older persons are associated with greater morbidity and mortality. Older individuals often present with atypical symptoms, such as lethargy, poor oral intake, or falls, rather than classical signs like fever or localized symptoms. This study aims to evaluate the clinical characteristics of older persons admitted with bacteraemia and identify predictors of in-hospital mortality.

**Methods:**

A retrospective observational cohort study was conducted at a tertiary hospital. Patients admitted to the geriatric ward with bacteraemia over a 1.5-year study period were included in the study. Demographic, clinical, and laboratory data were collected and analyzed to identify independent predictors for in-hospital mortality.

**Results:**

A total of 185 older patients were included in this study. Presentations were frequently non-specific, with 33.5% of the patients not fulfilling Systemic Inflammatory Response Syndrome (SIRS) criteria on admission. Advanced age (≥ 76 years old), hypalbuminaemia (< 20 g/L), elevated lactate (> 2 mmol/L), delirium, and absence of SIRS criteria were independently associated with increased in-hospital mortality of the older patients with bacteraemia.

**Conclusions:**

Due to immunosenescence and age-related physiological changes, older persons often present atypically and may have normal vital signs or laboratory values despite severe infection. Clinicians should maintain a high index of suspicion for sepsis in older patients, even in the absence of classical symptoms, to enable timely diagnosis and management.

## Introduction

Bacteraemia remains a major cause of morbidity and mortality in older persons, accounting for approximately 7% of infections in this population, with reported mortality rates of 11–30% within one week and up to 45% within one month [1–3]. Unlike younger adults, older individuals often present with atypical or muted manifestations of infection. Up to 30% may not develop fever, a key component of conventional diagnostic frameworks such as the Systemic Inflammatory Response Syndrome (SIRS) criteria [4, 5]. The absence of fever and other classical inflammatory responses has been associated with delays in diagnosis, postponed antimicrobial therapy, and increased mortality. In addition, decompensated comorbidities such as heart failure, chronic kidney disease, and chronic lung disease often obscure early recognition of bacteraemia in older patients.

Frailty further compounds the diagnostic and prognostic complexity of infection in older persons. Characterised by reduced physiological reserve and impaired resilience, frailty has been strongly associated with increased infection risk, prolonged hospitalisation, and mortality [6]. The Clinical Frailty Scale (CFS) provides a validated measure of frailty severity, with higher scores predicting poorer outcomes [7]. Frailty commonly coexists with immunosenescence, an age-related decline in immune function that attenuates inflammatory responses and weakens host defence. Together, frailty and immunosenescence create a state of heightened vulnerability, contributing to atypical clinical presentations and adverse outcomes in older persons with infection [8, 9]. These processes limit compensatory capacity during acute infection, rendering older persons vulnerable to abrupt clinical decline in the absence of classical inflammatory signs.

The clinical importance of bacteraemia in older persons is further amplified by ongoing population ageing. Globally, the population aged 60 years and above is projected to increase from 1 billion in 2020 to 1.4 billion by 2030. In Malaysia, the proportion of adults aged 65 years and above has risen steadily and is expected to reach 15% by 2040 [10]. Infectious diseases are currently ranked as the ninth leading cause of premature mortality among older persons in Malaysia [11]. Despite this, there remains a paucity of local data describing how bacteraemia presents in older persons and which clinical or biochemical features reliably predict poor outcomes in this setting.

Against this backdrop, there is a clear need to re-examine conventional sepsis recognition paradigms in older persons and to advocate for age-appropriate risk stratification. This study therefore aimed to evaluate the clinical characteristics of older persons admitted with bacteraemia to a geriatric ward and to identify clinical and biochemical predictors of in-hospital mortality. By highlighting factors associated with missed or delayed recognition and rapid deterioration, this study seeks to support earlier identification, more timely intervention, and improved outcomes for older persons with bacteraemia.

## Materials and methods

### Study population

This retrospective observational cohort study was conducted at the Universiti Malaya Medical Centre (UMMC), a tertiary hospital located in Kuala Lumpur, Malaysia. This study was approved by the UMMC Medical Research Ethics Committee (MREC ID: 2023405-1208). Consecutive patients with culture-proven bacteraemia during the study period (1 January 2018 to 30 June 2019) were identified from the Medical Microbiology Diagnostic Laboratory database. Eligibility criteria include patients aged ≥ 65 years who were admitted to the geriatric ward in UMMC with at least one positive blood culture during the index admission. Patients aged < 65 years or without positive blood cultures were excluded from the study.

### Clinical data collection

A standardised Case Report Form (CRF) was used to collect patients’ data retrieved from the hospital’s electronic medical records database. As this was a retrospective review of culture-proven bacteraemia, all eligible admissions were systematically identified, and complete clinical, laboratory, and outcome data were retrieved from the electronic medical records. There were no missing data for variables included in the analysis; therefore, no data imputation or case exclusion due to missing data was required.

Patients’ demographic and clinical data collected for this study include age, gender, comorbidities, pre-admission residences, pre-morbid ambulation status, vital signs and presenting symptoms during admission, sources of bacteraemia, type of organisms, length of hospital stay, mortality outcomes, and other pertinent information obtained through systematic chart review, including physician notes, admission and discharge documentation, and laboratory testing reports.

The sources of bacteraemia acquisition were categorised as community- or hospital-acquired based on isolation of bacteria from blood culture within 48 h of admission or beyond, respectively [12–14]. Comorbidities were quantified using the Charlson Comorbidity Index (CCI) [15]. Comprehensive geriatric assessments (CGA) were routinely performed on admission or within the first 48 h of hospitalisation. Frailty was assessed using the Clinical Frailty Scale (CFS) based on premorbid functional status, as documented by the attending geriatricians [6]. Delirium was systematically screened using My4AT, the Malaysian-validated version of the 4AT tool, which was administered daily by trained ward nurses and confirmed by attending geriatric physicians [16].

The SIRS criteria were determined based on Chakraborty and Burns’ (2023) definition [4]. The functional status of the patients was measured using basic and instrumental Activities of Daily Living (bADLs and iADLs) [17].

### Statistical analyses

Statistical analyses were performed using IBM SPSS Statistics (Version 23). Continuous variables were presented as mean ± standard deviation (SD) or median and interquartile range (IQR), whichever was appropriate. Categorical data were presented as counts (n) and frequencies (%). Sample size adequacy for multivariable analysis was assessed using the events-per-variable (EPV) rule. With 69 in-hospital mortality events, the number of covariates included in the multivariable Cox proportional hazards regression model adhered to accepted EPV thresholds. Variables selected for inclusion in the multivariable Cox regression model were chosen based on a combination of clinical plausibility, prior literature, and univariate associations, ensuring both statistical robustness and clinical interpretability. Lactate was modelled as a continuous variable in the regression analyses, while categorical cut-offs for age, albumin, and lactate were also applied based on established clinical thresholds to aid interpretability. Multicollinearity was assessed using variance inflation factors (VIF), with all VIF values < 2, indicating no significant collinearity between covariates.

The proportional hazards assumption of the Cox model was evaluated using Schoenfeld residuals and was not violated. Patients discharged alive were censored at the time of discharge. Variables found to be statistically significant in univariate analyses were entered into a Cox proportional hazards regression model to determine the independent predictors of in-hospital mortality. Results were reported as hazard ratios (HR) with 95% confidence intervals (95% CI). Kaplan–Meier survival curves were constructed to illustrate in-hospital mortality stratified by SIRS criteria. In all instances, statistical significance was indicated by *p* < 0.05.

## Results

### Baseline characteristics

A total of 185 eligible patients were identified from the hospital database and included in this study. Baseline characteristics of the patients are summarised in Table 1. The age of the patients ranged from 76 to 85 years old, with a mean age of 79.7 years (SD = 6.98). Both genders were equally represented in the patient population. The majority of the bacteraemia cases were associated with community-acquired infections (89.7%). Most of the patients stayed at family homes (72.4%) prior to the hospital admission. The majority of patients were bedbound (41.6%), and approximately half of them (47.0%) had pre-existing pressure sores upon admission. Most of the patients were dependent on their basic ADLs (69.2%), and all patients were moderately (7.0%) to severely frail (93.0%). Approximately three-quarters (74.1%) of the patients had high CCI (≥ 5), with hypertension, diabetes and stroke documented as the most prevalent comorbidities. One-third of the patients (31.9%) had delirium during admission. The median length of hospital stay was 15 days (IQR 8–21 days). More than half of the patients (62.7%) survived to discharge.

**Table 1:** Baseline characteristics of older patients (≥ 65 years) with bacteraemia (*n* = 185)

Characteristic	*n* (%)
Age (years) (mean ± SD)	79.7 ± 6.98
Age group (years)	
66–75	50 (27.0)
76–85	94 (50.8)
≥ 86	41 (22.2)
Gender	
Male	92 (49.7)
Female	93 (50.3)
Categories of bacteraemia acquisition	
Community-acquired	166 (89.7)
Hospital-acquired	19 (10.3)
Pre-admission residence	
Home	134 (72.4)
Nursing home	51 (27.6)
Premorbid ambulation status	
Independent	32 (17.3)
With aid	55 (29.7)
Wheelchair-bound	21 (11.4)
Bedbound	77 (41.6)
Pre-existing pressure sores on admission (stage I–IV)	87 (47.0)
Premorbid basic Activities of Daily Living (bADLs)	
Independent	57 (30.8)
Dependent	128 (69.2)
Clinical Frailty Scale (CFS) score	
Mild (4–5)	0 (0.0)
Moderate (6)	13 (7.0)
Severe (7–9)	172 (93.0)
Charlson Comorbidity Index (CCI) severity	
Mild (1–2)	1 (0.5)
Moderate (3–4)	47 (25.4)
Severe (≥ 5)	137 (74.1)
Delirium during admission	59 (31.9)
Length of hospital stay (days), median (IQR)	15 (8–21)
Discharge status	
Alive	116 (62.7)
Died	69 (37.3)

### Clinical and microbiological characteristics

Approximately two-thirds of the older patients (66.5%) fulfilled SIRS criteria, presenting at least two of the four parameters (Table 2). The majority of the patients (69.7%) had abnormal white blood cell counts (< 4 or > 12 × 10⁹/L), and 68.6% of the patients had elevated heart rate (> 100 bpm). However, only one-third of the patients had altered body temperature (≥ 38 °C or ≤ 36 °C) (30.3%) and respiratory rate of more than 20 breaths per minute (28.6%). Fever was reported in only 27.0% of the bacteraemia cases. Approximately half of the patients (47.6%) presented with non-specific symptoms such as confusion, lethargy, or reduced responsiveness. Other less common complaints (18.9%) included seizures, falls, diarrhoea, abdominal pain, and shortness of breath.

**Table 2:** Clinical and Microbiological characteristics of older patients with bacteraemia

Characteristic	*n* (%)
SIRS criteria fulfilled (*n* = 123)	79.7 ± 6.98
White blood cell count abnormality	129 (69.7)
Tachycardia (heart rate > 90/min)	127 (68.6)
Fever/hypothermia (temperature criterion)	56 (30.3)
Tachypnea (respiratory rate > 20/min)	53 (28.6)
Presenting complaints (*n* = 185)	
Altered mental status (less responsive / unwell / lethargy / confusion)	88 (47.6)
Fever	50 (27.0)
Other complaints	35 (18.9)
Microbiological characteristics (*n* = 185)	
Monomicrobial bacteraemia	174 (94.1)
Polymicrobial bacteraemia	11 (5.9)
Gram-negative organisms	117 (63.2)
Gram-positive organisms	66 (35.7)
Other organisms	2 (1.1)

Respiratory tract infections were the predominant source of bacteraemia in this cohort of older patients (37.8%), followed by urinary tract infections (27.6%) and skin infections (10.8%). Other sources include intra-abdominal infections and infections related to central/vascular catheterisation (23.8%).

Almost all patients (94.1%) had monomicrobial bacteraemia, predominated by Gram-negative organisms (63.2%) (Table 2). Candidaemia was identified in 1.1% of the cases. The most frequently isolated Gram-negative organism was *Escherichia coli* (48.7%), followed by *Klebsiella pneumoniae* (25.6%), *Proteus mirabilis* (9.4%), and *Pseudomonas aeruginosa* (5.1%). Less commonly identified Gram-negative organisms included *Morganella morganii*, *Acinetobacter baumannii*, *Enterobacter* spp., *Bacteroides* spp., *Achromobacter xylosoxidans*, and *Fusiform* bacteria, collectively contributing to 11.1% of the bacteraemia cases. Among the isolated Gram-positive organisms (*n* = 66), methicillin-resistant coagulase-negative staphylococci (MRCONS) accounted for the highest proportion (34.8%), followed by methicillin-sensitive *Staphylococcus aureus* (MSSA; 31.8%) and *Streptococcus pneumoniae* (3.0%). Less commonly detected Gram-positive organisms included *Corynebacterium* spp., *Globicatella* spp., *Enterococcus* spp., *Bacillus cereus*, *Dolosigranulum pigrum*, *Eggerthella lenta*, *Peptoniphilus asaccharolyticus*, and *Kocuria kristinae*, isolated from one-third of the bacteraemia patients (30.3%).

### Clinical and laboratory markers for sepsis

**Fig. 1:**
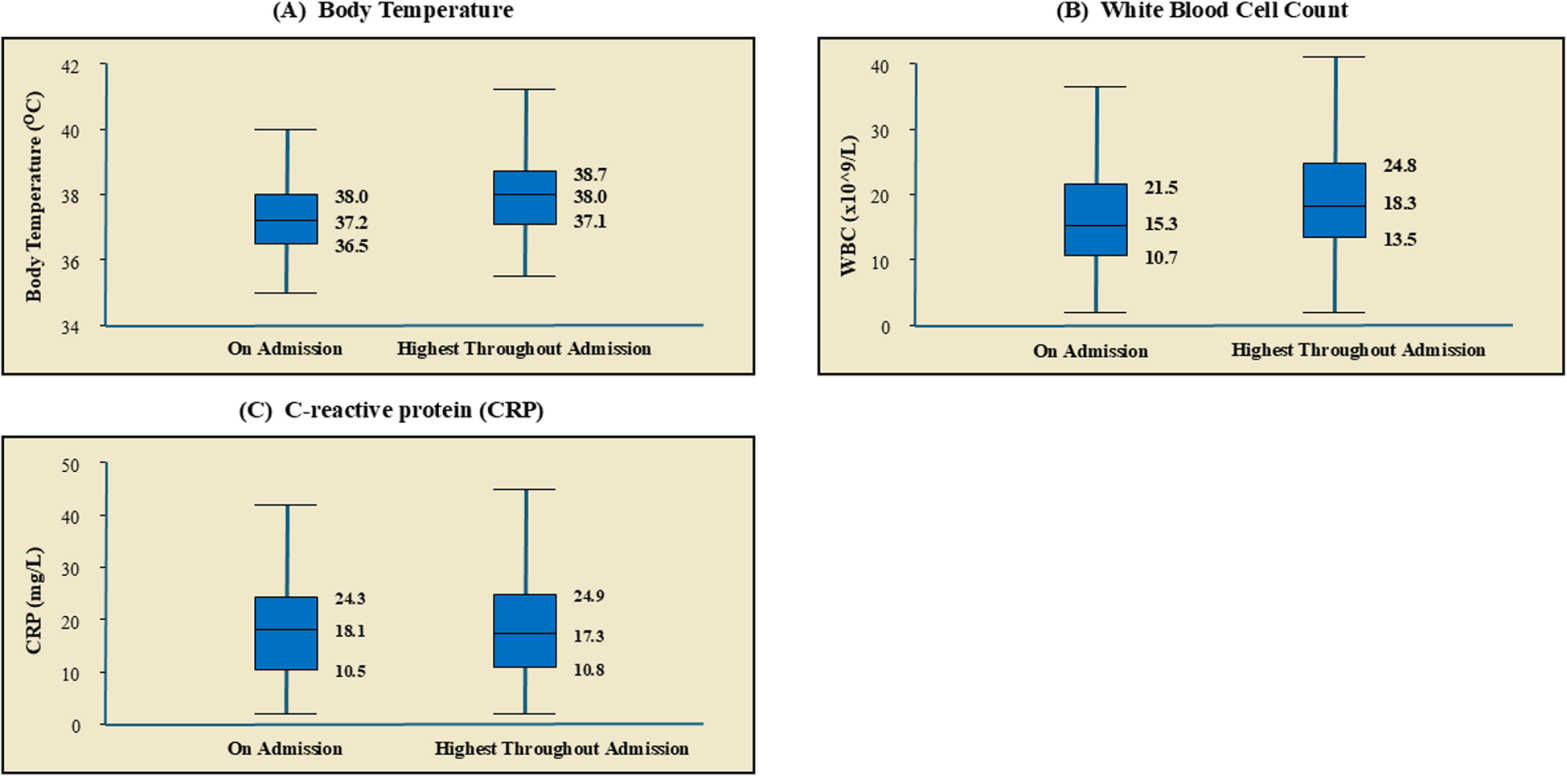
Distribution of (**A**) Body Temperature, (**B**) White Blood Cell count, and (**C**) CRP (median and interquartile ranges) on admission and at peak values during hospitalisation

On admission, the median body temperature of the patients was below 38 °C (IQR 36.5–38.0), with a peak median of 38 °C observed on days 3–4, suggesting a delayed systemic inflammatory response. The median white blood cell (WBC) count at admission was 15.3 × 10⁹/L (IQR 10.7–21.5), peaking later during days 2–4 at 18.3 × 10⁹/L (IQR 13.5–24.8), further reflecting a delayed response. The median admission value for C-reactive protein (CRP) was 18.1 mg/L (IQR 10.5–24.3), with subsequent levels remaining within a similar range, indicating limited dynamic change over the course of admission (Fig. 1). 

### Predictors for In-hospital mortality

**Fig. 2:**
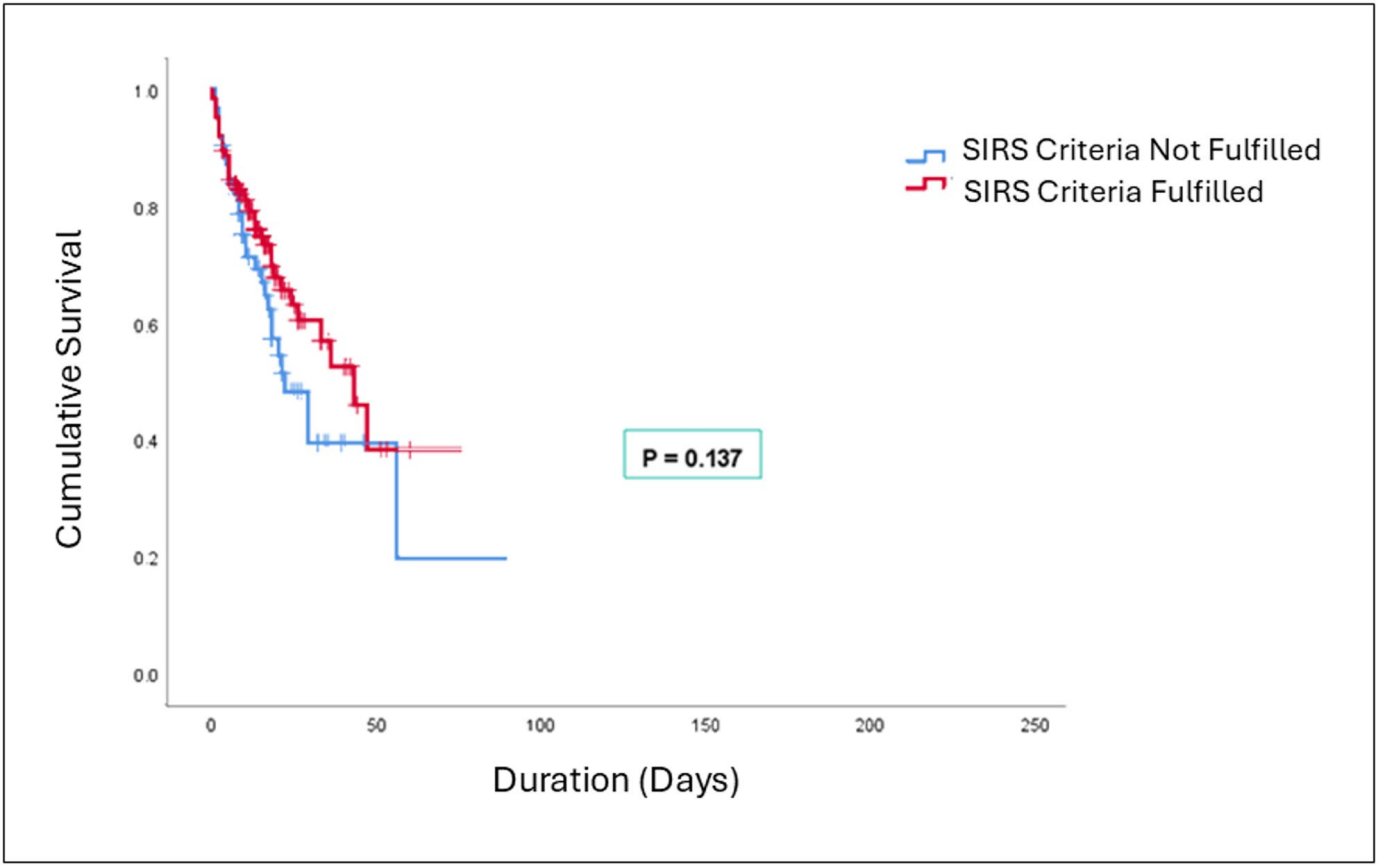
Kaplan–Meier survival curve for in-hospital mortality among older patients with bacteraemia stratified by fulfilment of the SIRS criteria on admission

The older patients who did not fulfil the SIRS criteria at hospital admission demonstrated lower survival rates compared to those who fulfilled the criteria (*p* = 0.137) (Fig. 2). Cox univariate regression analysis was conducted to identify risk factors associated with in-hospital mortality among older patients admitted with bacteraemia. Factors significantly associated with in-hospital mortality (*p* < 0.05) on univariate analysis included age group, gender, serum lactate, serum albumin, white blood cell count, fulfilment of the SIRS criteria, delirium, and Gram-negative bacteraemia, and several of these were identified as independent predictors on subsequent multivariate Cox regression analysis (Table  3). Patients aged ≥ 76 years had approximately twice the hazard of in-hospital mortality compared with those aged 66–75 years (HR 2.0, 95% CI 1.08–3.73; *p* = 0.028). Each 1 mmol/L increase in serum lactate above 2 mmol/L was associated with a 20% increase in the hazard of death (HR 1.2, 95% CI 1.09–1.26; *p* < 0.001). Hypoalbuminemia (< 20 g/L) was independently associated with higher mortality (HR 1.8, 95% CI 1.01–3.07; *p* = 0.047). The presence of delirium during admission also conferred a higher hazard of death (HR 1.3, 95% CI 1.07–3.52; *p* = 0.049). Interestingly, patients who did not fulfil SIRS criteria on admission had a significantly increased hazard of mortality (HR 1.9, 95% CI 1.10–3.42; *p* = 0.022).

**Table 3:** Risk factors associated with in-hospital mortality among older patients with bacteraemia (*n* = 185)

Variable	Crude HR (95% CI)	*P* value	Adjusted HR (95% CI)	*P* value
Age group (years)				
66–75	1 (reference)	–	1 (reference)	–
≥ 76	1.8 (1.08–3.08)	0.026*	2.0 (1.08–3.73)	0.028*
Gender				
Male	1 (reference)	–	–	–
Female	0.7 (1.44–2.15)	0.045*	–	–
Lactate	1.2 (1.08–1.24)	< 0.001*	1.2 (1.09–1.26)	< 0.001*
Albumin (≤ 20 g/L)	1.7 (1.00–2.75)	0.048*	1.8 (1.01–3.07)	0.047*
White blood cell count (outside 4–10 × 10⁹/L)	1.5 (0.89–2.66)	0.038*	–	–
Temperature	1.0 (0.83–1.20)	0.994	–	–
C-reactive protein (CRP)	1.0 (0.99–1.01)	0.825	–	–
Delirium	1.3 (1.09–3.61)	0.048*	1.3 (1.07–3.52)	0.049*
SIRS criteria fulfilled	1.7 (1.43–2.13)	0.043*		
Yes	1 (reference)	–	1 (reference)	–
No	–	–	1.9 (1.10–3.42)	0.022*
Clinical Frailty Scale				
Moderate	1 (reference)	–	–	–
Severe	1.5 (0.47–4.76)	0.496	–	–
Charlson Comorbidity Index	1.1 (1.00–1.21)	0.064	–	–
Gram-negative bacteraemia	0.7 (0.44–1.14)	0.039*	–	–

Adjusted for age group, serum lactate, serum albumin, delirium, and SIRS criteria

*HR* Hazard ratio, *CI* Confidence interval, *SIRS* Systemic Inflammatory Response Syndrome criteria

* *p* < 0.05 indicates statistical significance

## Discussion

This study demonstrates that bacteraemia in older persons admitted to geriatric ward is characterised by atypical clinical presentation and substantial in-hospital mortality, with several readily available clinical and biochemical factors independently associated with adverse outcomes. In this predominantly frail, multimorbid cohort, over one-third of patients died during admission, and a significant proportion did not fulfil SIRS criteria at presentation, despite culture-proven bloodstream infection. Advanced age (≥ 76 years old), hypalbuminaemia, elevated serum lactate, delirium, and notably the absence of SIRS criteria on admission emerged as independent predictors of in-hospital mortality. Collectively, these findings highlight a critical mismatch between conventional sepsis recognition frameworks and the physiological reality of older persons, in whom immunosenescence, frailty, and altered stress responses may mask classical inflammatory signals while conferring a heightened risk of death. This frailty distribution reflects the real-world case-mix of tertiary geriatric wards rather than selection bias. Our results therefore reinforce the need to reconsider reliance on traditional sepsis markers in geriatric populations and to adopt a more nuanced, age-informed approach to early risk stratification and clinical decision-making.

The SIRS criteria have been widely used in early goal-directed therapy (EGDT) to identify sepsis [18]. Prior studies have linked positive SIRS results with increased risk of adverse outcomes and mortality in patients [19, 20]. However, despite its high sensitivity for early risk assessment, only 66.5% of the older patients in this study met the SIRS criteria upon presentation. Increased heart rate and abnormal white blood cell count were two of the SIRS parameters that were more commonly detected in our study population. Only one-third of the older patients with bacteraemia met the altered body temperature and respiratory rate criteria. Indeed, a delayed response was observed in the patients’ body temperature rise and white blood cell count, with both parameters peaking two to three days post-admission. Although survival differences did not reach statistical significance, patients who did not fulfil SIRS criteria demonstrated a trend toward poorer survival, indicating that apparent physiological “normality” at presentation may reflect blunted host responses rather than clinical stability.

Our findings support growing evidence that the sensitivity of SIRS criteria is reduced in older adults, likely reflecting age-related physiological changes and immunosenescence. In frail older persons, failure to meet SIRS thresholds may therefore represent vulnerability and delayed recognition rather than lower illness severity. These observations reinforce the need for age-adapted approaches to sepsis assessment that extend beyond traditional inflammatory markers and prioritise early identification of occult physiological derangement.

Older persons frequently present with atypical clinical manifestations of infection, including delirium, functional decline, and other non-localising symptoms, which complicates early recognition of bacteraemia. In this study, more than half of patients presented with non-specific symptoms at admission; however, only 32% were formally diagnosed with acute delirium, suggesting that its true incidence was likely underestimated. Delirium has been well recognised as a marker of acute physiological stress and vulnerability in older persons and is consistently associated with adverse outcomes, including mortality, prolonged hospitalisation, and functional decline [21–25]. Consistent with prior literature, delirium in our cohort was independently associated with increased in-hospital mortality, conferring an approximately 30% higher hazard of death.

Several age-related physiological mechanisms may explain both the atypical presentation of infection and the high burden of delirium observed in older patients. Fever, a classical hallmark of infection, may be blunted due to altered thermoregulation, including impaired sudomotor and vasomotor responses and reduced production or responsiveness to endogenous pyrogens such as IL-1, IL-6, and TNF [26, 27]. Previous studies have demonstrated that older patients frequently present without fever despite having bacteraemia [28–31]. Similarly, heart rate responses may be attenuated by beta-blocker therapy and age-related cardiovascular changes, including reduced pacemaker cell numbers, fatty infiltration of conduction fibres, and decreased sinus automatism [32–35]. Age-related alterations in respiratory mechanics further obscure clinical detection, as structural changes of the thoracic cage, reduced chest wall compliance, diaphragmatic sarcopenia, and decreased intercostal muscle mass diminish ventilatory reserve, while blunted dyspnoea perception and reduced ventilatory responses to hypoxia and hypercapnia increase vulnerability to respiratory failure [36–39].

In addition to age-related physiological changes, immunosenescence further contributes to atypical laboratory responses in older patients. Thymic involution reduces naïve T-cell production, leading to impaired adaptive immunity, while functional deficits in macrophages, neutrophils, and natural killer cells compromise innate immune responses [40–44]. These changes likely underpin the observed blunted SIRS responses and altered white blood cell dynamics among the older patients with bacteraemia. Consistent with this, C-reactive protein (CRP) demonstrated minimal dynamic variation and was not associated with in-hospital mortality in this cohort. As CRP production is largely IL-6 dependent, age-related cytokine dysregulation and frailty-associated immune exhaustion may partially explain its poor discriminative performance in this population. This likely reflects reduced hepatic responsiveness to interleukin-6 and other pro-inflammatory mediators, a hallmark of immunosenescence, rather than the absence of infection severity per se. Contemporary observational data indicate that older, frail patients may mount blunted CRP responses despite serious infection, which undermines CRP’s prognostic utility for in-hospital mortality in bacteraemic contexts [45, 46]. The diagnostic and prognostic value of CRP must be interpreted in the wider clinical context, particularly in elderly cohorts where frailty, comorbidity, and dysregulated inflammation influence biomarker performance. Narrative reviews on sepsis biomarkers emphasize that while CRP remains a widely available marker, its predictive accuracy for mortality is enhanced when used alongside other biomarkers and clinical scoring systems, though performance may still be constrained by age-related changes in immune signalling [46]. These findings caution against reliance on CRP trends alone for risk stratification in geriatric sepsis and support greater emphasis on physiological vulnerability markers such as delirium, lactate, and functional reserve.

In our study cohort, patients who did not fulfil SIRS criteria had a significantly higher hazard of in-hospital mortality (HR 1.9; 95% CI 1.10–3.42; *p* = 0.022). This finding supports prior observations that conventional sepsis criteria may underperform in older adults. Rather than recommending formal integration of the scoring system, our findings raise the hypothesis that combined or sequential use of SIRS and qSOFA may warrant prospective evaluation in older populations, particularly those with high frailty burden. Such an approach may help balance sensitivity and specificity for early sepsis detection, support timely clinical decision-making, and optimize resource allocation. Future research should explore the utility of combined SIRS and qSOFA scoring to determine whether it enhances risk stratification for older adults with bacteraemia.

Advanced age (≥ 76 years old), hypoalbuminemia (< 20 g/L), and elevated lactate (> 2 mmol/L) were among the independent predictors of in-hospital mortality for our patients cohort. These findings corroborate previous reports linking advanced age, frailty, immunosenescence, and comorbidities to poor sepsis outcomes [47–51]. Albumin is a well-established prognostic marker in sepsis, reflecting systemic inflammation, capillary leak, and oxidative stress [52–54]. Elevated lactate is similarly recognized as a sensitive indicator of tissue hypoperfusion and mortality risk [55–57].

Although frailty is a well-established predictor of adverse outcomes in infection, the CFS did not retain independent significance in multivariable analysis in this study. This is likely attributable to restricted variability and a ceiling effect, as over 90% of patients were classified as severely frail. In such a uniformly frail population, frailty may function as a background vulnerability rather than a discriminating predictor of mortality. Rather than diminishing its clinical relevance, this finding underscores that frailty characterised the entire cohort and likely modified the impact of acute physiological insults such as lactataemia, delirium, and hypoalbuminaemia. Delirium serves as a clinical marker of physiologic vulnerability conferred by frailty, and delirium risk escalates with advancing frailty and comorbidity burden [58]. In frail individuals, diminished hepatic and mitochondrial reserve may alter lactate production and clearance, potentially intensifying or prolonging acid-base disturbances during acute illness or shock. The literature on lactic acidosis in frailty explicitly links metabolic derangements to reduced physiologic reserve and poorer outcomes, underscoring frailty as a modifier of the prognostic significance of lactate levels in acute settings [59]. The frail state often accompanies malnutrition and chronic inflammation, creating a milieu in which hypoalbuminemia reflects diminished hepatic synthetic capacity and catabolic stress. This milieu aggravates vulnerability to acute insults and complicates recovery and outcomes [60]. Hence, frailty profoundly modulates the clinical impact of acute physiological insults such as lactic acidosis, delirium, and hypoalbuminemia. The diminished physiological reserve inherent to frailty amplifies vulnerability to metabolic stress and neurocognitive disturbance, contributing to worse outcomes, including mortality and functional decline. Accordingly, CFS was retained in univariate analysis and assessed for inclusion but was excluded from the final multivariable model to preserve model stability and avoid overfitting in the context of limited frailty dispersion.

We acknowledge the potential influence of unmeasured confounders, such as prior hospitalizations and timing of antibiotic administration, on the observed outcomes. Additionally, frailty severity may act as an effect modifier, potentially altering the relationship between clinical variables (e.g., SIRS criteria, delirium) and in-hospital mortality in older persons.

The findings reported in this study should be interpreted in light of several limitations. First, the relatively small sample size and retrospective nature of this single centre study may limit the generalizability of the results. Second, our study cohort predominantly comprised severely frail and bed-bound patients, which may have led to an over-representation of frailty-related outcomes and an under-representation of healthier older persons. Third, microbiological data did not include detailed antimicrobial resistance profiles, preventing evaluation of the impact of resistance patterns on outcomes. Nonetheless, this study offers important insights into the epidemiology, clinical presentation, and outcomes of bacteraemia in older persons in Malaysia.

## Conclusions

Older persons with infections frequently present with atypical clinical features and blunted physiological responses due to immunosenescence and frailty, which can delay both diagnosis and the initiation of antimicrobial therapy. In this study, advanced age (≥ 76 years), elevated lactate (> 2 mmol/L), hypoalbuminaemia (< 20 g/L), delirium, and absence of SIRS criteria were independently associated with in-hospital mortality. Clinicians should maintain a high index of suspicion for sepsis in older patients, even in the absence of classical signs, to enable timely recognition and management. These findings highlight the need to reconsider reliance on conventional SIRS criteria alone and to adapt sepsis assessment approaches to better reflect older persons’ physiology. Future research should prioritise prospective validation of alternative or combined risk stratification tools specifically tailored to geriatric populations, with systematic inclusion of frailty and delirium assessments. Educating healthcare professionals on the nuances of infection in older persons is essential to improve outcomes and ensure effective care.

## Data Availability

All data generated or analysed during this study are included in this published article.

## Abbreviations

SIRS: Systemic Inflammatory Response Syndrome
CFS: Clinical Frailty Scale
UMMC: Universiti Malaya Medical Centre
MREC: Medical Research Ethics Committee
CRF: Case Report Form
CCI: Charlson Comorbidity Index
BADLs: Basic Activities of Daily Living
IADLs: Instrumental Activities of Daily Living
SD: Standard deviation
IQR: Interquartile range
HR: Hazard ratios
CI: Confidence intervals
MRCONS: Methicillin-Resistant Coagulase-Negative Staphylococci
MSSA: Methicillin-Sensitive Staphylococcus aureus
WBC: White blood cell
CRP: C-reactive protein
EGDT: Early goal-directed therapy
IL-1: Interleukin-1
IL-6: Interleukin-6
TNF: Tumor Necrosis Factor
qSOFA: Quick Sequential Organ Failure Assessment
